# Anthocyanin Absorption and Metabolism by Human Intestinal Caco-2 Cells—A Review

**DOI:** 10.3390/ijms160921555

**Published:** 2015-09-08

**Authors:** Senem Kamiloglu, Esra Capanoglu, Charlotte Grootaert, John Van Camp

**Affiliations:** 1 Laboratory of Food Chemistry and Human Nutrition (nutriFOODchem), Department of Food Safety and Food Quality, Faculty of Bioscience Engineering, Ghent University, Coupure Links 653, B-9000 Ghent, Belgium; E-Mails: senem.kamiloglu@ugent.be (S.K.); charlotte.grootaert@ugent.be (C.G.); john.vancamp@ugent.be (J.V.C.); 2 Department of Food Engineering, Faculty of Chemical and Metallurgical Engineering, Istanbul Technical University, 34469 Maslak, Istanbul, Turkey

**Keywords:** anthocyanins, Caco-2 cells, intestinal absorption, metabolism

## Abstract

Anthocyanins from different plant sources have been shown to possess health beneficial effects against a number of chronic diseases. To obtain any influence in a specific tissue or organ, these bioactive compounds must be bioavailable, *i.e*., effectively absorbed from the gut into the circulation and transferred to the appropriate location within the body while still maintaining their bioactivity. One of the key factors affecting the bioavailability of anthocyanins is their transport through the gut epithelium. The Caco-2 cell line, a human intestinal epithelial cell model derived from a colon carcinoma, has been proven to be a good alternative to animal studies for predicting intestinal absorption of anthocyanins. Studies investigating anthocyanin absorption by Caco-2 cells report very low absorption of these compounds. However, the bioavailability of anthocyanins may be underestimated since the metabolites formed in the course of digestion could be responsible for the health benefits associated with anthocyanins. In this review, we critically discuss recent findings reported on the anthocyanin absorption and metabolism by human intestinal Caco-2 cells.

## 1. Introduction

Anthocyanins are water-soluble pigments responsible for the blue, purple, and red color of many plant tissues [1]. The term anthocyanin is derived from the Greek words *anthos*, meaning flower, and *kyanos*, meaning blue [2]. Although they also occur in vegetables, roots, legumes, and cereals, these pigments are usually associated with fruits. In particular, berry fruits are rich sources of dietary anthocyanins [3,4,5,6] and can contribute tens to hundreds of milligrams of anthocyanins in a single serving. The daily intake of anthocyanins in the USA diet is estimated to be as much as 180–255 mg per day; a value that far exceeds the consumption of most other flavonoids [7].

Anthocyanins have become increasingly important to the food industry, as their use as natural alternatives to synthetic dyes has become widespread [8]. According to the numbering system used by the *Codex Alimentarius Commission*, anthocyanins are listed as a natural colorant by the EU (European Union) legislation and coded as E163. With respect to the USA, the FDA (Food and Drug Administration) has a different list of “natural” colors that do not require certification, and anthocyanins can be obtained either from “grape color extract”, “grape skin extract”, or “fruit or vegetable juices” [9].

Apart from their colorant features, many studies have associated anthocyanins with antioxidant, anti-inflammatory and anticarcinogenic properties, protection against both heart disease and certain types of cancer, as well as a reduction in the risk of diabetes and cognitive function disorders [10]. The potential availability of anthocyanins after gastrointestinal digestion is important, since a poor bioavailability of a certain anthocyanin would lead to a limited effect on health. One of the key factors affecting the bioavailability of anthocyanins is their transport through the gut epithelium, which can be investigated using *in vivo* studies and *in vitro* models. The Caco-2 cell line, a human intestinal epithelial cell model derived from a colon carcinoma, has been proven to be a good alternative to animal studies for predicting intestinal absorption of anthocyanins [11]. In this perspective, this review summarizes the recent findings reported on the absorption and metabolism of anthocyanins by human intestinal Caco-2 cells.

## 2. Chemistry of Anthocyanins

Anthocyanins belong to a large group of compounds collectively known as flavonoids, which are a subgroup of an even larger group of compounds known as polyphenols [7]. Chemically, anthocyanins occur as glycosides of flavylium (2-phenylbenzopyrylium) salts but differ from them by structural variations in the number of hydroxyl groups, the degree of methylation of these hydroxyl groups, the nature and number of sugar moieties attached to the molecule, and the position of the attachment, as well as the nature and number of aliphatic or aromatic acids attached to the sugars [12].

**Figure 1:**
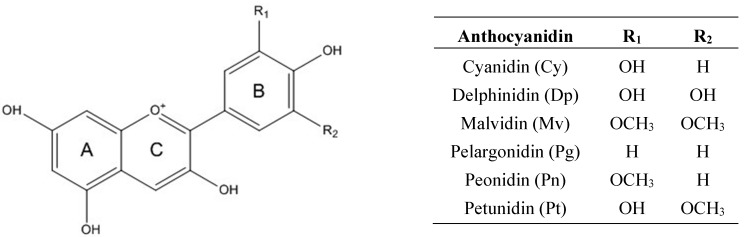
Anthocyanidin structures.

Anthocyanins are found as glycosides of their respective aglycones, called anthocyanidins [13]. The anthocyanidins consist of an aromatic ring A bound to a heterocyclic ring C that contains oxygen, which is also bound by a carbon–carbon bond to a third aromatic ring B [14]. About 17 anthocyanidins have been identified, but only six of them are commonly distributed in nature: cyanidin (Cy), delphinidin (Dp), malvidin (Mv), pelargonidin (Pg), peonidin (Pn) and petunidin (Pt) (Figure 1). Despite there being only six common anthocyanidins, there are over 600 anthocyanins reported in plants [15]. Glucose (Glu), galactose (Gal), arabinose (Ara), rutinose (Rut), rhamnose (Rham), and xylose (Xyl) are the most common sugars that are bound to anthocyanidins as mono-, di-, or trisaccharide forms [16]. The most widespread glycoside derivatives in nature are 3-monosides, 3-biosides, 3,5- and 3,7-diglucosides. The presence of the 3-glucoside derivatives is 2.5 times more frequent than the 3,5-diglucosides and the most common anthocyanin is Cy-3-Glu [17]. In many cases, the sugar residues are acylated with *p*-coumaric, caffeic, ferulic, sinapic, *p*-hydroxybenzoic, malonic, oxalic, malic, succinic or acetic acid [18].

**Figure 2:**
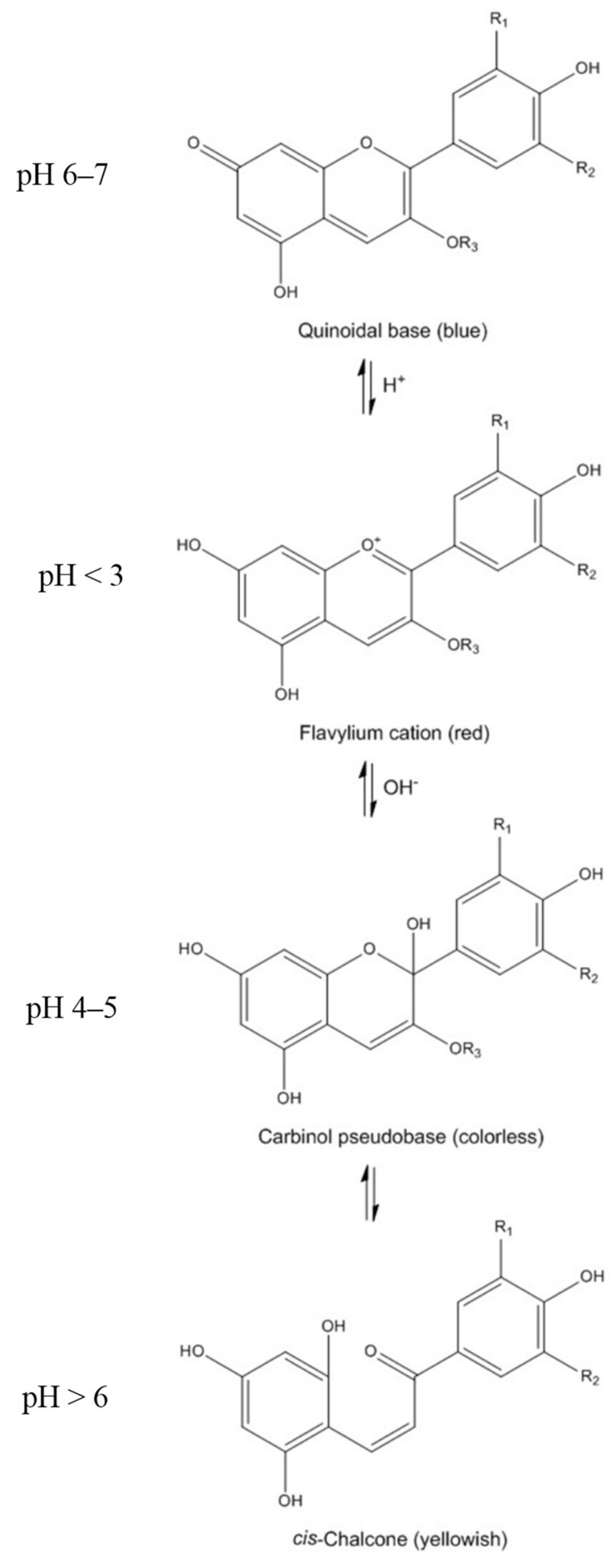
Molecular structures of anthocyanins under different pH conditions.

Anthocyanins are highly instable and very susceptible to degradation. Oxygen, temperature, light, enzymes and pH are among the many factors that may affect the chemistry of anthocyanins and, consequently, their stability and color [19]. The hue of anthocyanins may vary according to different substituent groups present on the B ring, and color saturation increases with increasing number of hydroxyl groups and decreases with the addition of methoxyl groups [20]. In aqueous solution, anthocyanins undergo structural re-arrangements in response to changes in pH in four molecular structures: quinoidal base (blue), flavylium cation (red), carbinol (colorless) and chalcone (yellowish) forms (Figure 2). Anthocyanins are stable in acidic solutions (pH 1–3) where they exist primarily as flavylium cations. At pH > 4, anthocyanins adopt the forms of the carbinol and chalcone. Chalcone can then undergo chemical degradations to produce phenolic acids [16]. The relative composition of the different molecular structures of anthocyanins coexisting in aqueous solution at any given time will depend on pH, temperature and time. This is particularly important as anthocyanins are exposed to different pH conditions through the gastrointestinal tract, which affects their bioavailability and hence their bioactivity [7].

## 3. Bioavailability of Anthocyanins: Absorption and Metabolism by Caco-2 Cells

Bioavailability is defined by the FDA as “rate and extent to which the active ingredient or moiety is absorbed and becomes available at the site of action” [21]. Methods for determination of bioavailability of anthocyanins include human (*in vivo*) or simulated experiments performed in a laboratory (*in vitro*). *In vivo* methods provide direct data of bioavailability and have been used for a large variety of nutrients. On the other hand, *in vitro* methods have the advantage of being more rapid, less expensive, less labor intensive, and do not have ethical restrictions. *In vitro* methods simulate gastrointestinal digestion under controlled conditions using commercial digestive enzymes, whereas the final absorption process is commonly assessed using Caco-2 cell cultures [22]. Here, we will focus on anthocyanin transport and metabolism through Caco-2 cells. For a more comprehensive overview on bioavailability aspects of anthocyanins, please refer to these other reviews [7,16,19].

### 3.1. Caco-2 Cell Growth and Differentiation

The Caco-2 cell line has been established by Fogh and co-workers in 1977 from a human colon adenocarcinoma, and originally used for the screening of cytotoxic effects of anti-tumor drugs and for the study of drug resistance mechanisms [23]. During the past few decades, this cell line has been extensively used for cellular permeability studies of polyphenols [24,25,26,27]. It has been well established that Caco-2 cells can undergo spontaneous differentiation in culture conditions and exhibit the characteristics of mature enterocytes. The cell surface facing the top medium develops a brush border that resembles the luminal membrane of the intestinal epithelium. The cell surface attaching to the permeable membrane and facing the bottom medium develops into the basolateral membrane [28,29]. Despite their colonic origin, Caco-2 cells express the morphological and functional characteristics of small intestinal cells. The Caco-2 monolayer houses multiple transporters, receptors and metabolic enzymes such as cytochrome P450 1A (CYP1A), sulfotransferases (SULTs), UDP-glucuronosyltransferases (UGTs), and glutathione S-transferases (GSTs) [30].

Transport experiments are generally carried out using filter-based inserts, where cells are seeded and allowed to grow and differentiate to confluent monolayers for approximately 21 days post seeding. Before performing the transport experiment, the integrity of the Caco-2 monolayer is controlled by measuring the transepithelial electrical resistance (TEER), or, more reliably, by examining the permeability of paracellular markers such as mannitol, inulin, Dextran, PEG 4000, and lucifer yellow [30]. TEER is a non-invasive technique, which measures the impedance between the lumen and basolateral tissue. TEER measurements use a constant direct current applied by two electrodes, one connected with the lumen side and the other one with the basolateral side. By applying Ohm’s law it is possible to measure the related cells resistance [31]. It was reported in the literature that an acceptable TEER value for Caco-2 cell monolayers should be from 200 to 1000 ohm per cm^2^ [32]. Since its original isolation, the Caco-2 cell line has been propagated in several laboratories around the world, producing Caco-2 cells of different “age”, or number of passages in culture. The expressions of typical differentiation markers of intestinal enterocytes were shown to increase from early to late passages. Accordingly, the TEER value has also been demonstrated to increase in later passages of cell monolayer [33].

In order to reduce the heterogeneity of the Caco-2 parental cell line and to improve the performance and the stability of this cellular model, some clonal cell lines have been obtained from Caco-2. Among them, TC-7 is often used to simulate polyphenol transport [34,35,36]. The TC-7 clone exhibited similar cell morphology to Caco-2 cells, displaying the presence of brush-border membrane and microvilli, and the formation of tight junctions. Similarly, on the basis of biochemical attributes and permeability characteristics, the TC-7 subclone appears to be similar to Caco-2 cells and presents a suitable alternative to parental cells for intestinal permeability studies [37].

The HT-29 cell line is another cell line from colorectal origin with epithelial morphology, and has been used as a model for absorption, secretion and transport by intestinal cells. Under standard culture conditions, these cells grow as a non-polarized, undifferentiated monolayer. However, altering culture conditions or treating the cells with different inducers results in a differentiated and polarized morphology, characterized by a redistribution of membrane antigens and development of an apical brush-border membrane [38]. Other human intestinal cell lines are less popular for the simulation of the human intestinal epithelium, such as the HCT-116 and SW480 cell lines, which are mainly used in unraveling cancer-related mechanisms [39], and the HuTu-80 cell line, a model for duodenal cells [40].

### 3.2. Anthocyanin Transport through Caco-2 Cells

Studies investigating anthocyanin absorption by Caco-2 cells are presented in Table 1 [11,28,29,41,42,43,44,45,46,47,48,49]. The majority of these studies suggest that unlike other flavonoids, anthocyanins could be transported through Caco-2 monolayers in intact glycone forms, with the exceptions of black currant and some grape anthocyanins. Steinert *et al*. [41] demonstrated that anthocyanins from black currant, namely Dp-3-Glu, Dp-3-Rut, Cy-3-Glu and Cy-3-Rut, were not detected in any serosal solution. However, the authors showed that anthocyanins disappeared from the luminal side, not due to the anthocyanin degradation process but rather due to physiological actions of the cells. Similarly, Dp-3-Glu from grape extract was not transported [48]. Diglucosylated Mv-3,5-DGlu and Pn-3,5-DGlu from grape/blueberry extract were also not transported in quantifiable concentrations [49]. On the other hand, studies that observed anthocyanin transport, reported very low transport efficiencies. The transport efficiency of anthocyanins from blueberry extracts averaged *ca*. 3%–4% (<1% in Dp-3-Glu) [28]. Similarly, only about 1% of the red grape skin anthocyanins passed through a Caco-2 cell monolayer and reached the basolateral side [42]. The percentage of transported monomeric anthocyanin glycosides from açaí fruit ranged from 0.5% to 4.9% [43], whereas according to Cardona *et al*. [46] the transport rate of açaí anthocyanins was 1.2%. Transport efficiencies of Mv-3-Glu and Cy-3-Glu standards were found to be 4% and 0.8%–2.4%, respectively [44,45]. Moreover, Cy-3-Glu-Rut recovery from sour cherry fruit and nectar was *ca*. 0.5%–4% [11]. Trace amount of Pg-3-Glu, the predominant anthocyanin from strawberry extract, was found on the basolateral side of the epithelium [47]. Transport efficiency of the major grape anthocyanin (Mv-3-Glu) was 0.35% [48], while the absorption rates of Mv-3-Glu, Pn-3-Glu, Pt-3-Glu, Dp-3-Glu and Cy-3-Glu from grape/blueberry extract were 0.005%–0.06% [49]. These results are in line with *in vivo* studies showing a very low bioavailability of anthocyanins, with <1% of the ingested amount reaching the plasma or being excreted in the urine [50,51,52,53,54].

**Table 1:** Studies investigating anthocyanin absorption by Caco-2 cells.

Sample	Pre-Treatment	Anthocyanins	Anthocyanin Concentration	Cell Origin	Cell Differentiation	Incubation Time	Key Findings	Reference
Blueberry	Chemical extraction	Dp-3-Glu, Cy-3-Gal, Cy-3-Glu, Pt-3-Glu, Pn-3-Gal, Pn-3-Glu, Mv-3-Glu	50 μg/mL	ATCC	20–26 days	0–120 min	Transport efficiency of ACNs averaged *ca*. 3%–4% (<1% in Dp-3-Glu); Glucose-based ACNs had higher bioavailability than galactose-based ACNs	[28]
Black currant extract	-	Dp-3-Glu, Dp-3-Rut, Cy-3-Glu, Cy-3-Rut	180 μM	DSWZ	19–21 days	0–80 min	ACNs were not detected in any serosal solution	[41]
Red grape skin	Chemical extraction	Dp-3-Glu, Cy-3-Glu, Pt-3-Glu, Pn-3-Glu, Mv-3-Glu	200 μg/mL	ATCC	25 days	4 days of pre-treatment + 6 min	Only *ca*. 1% of ACNs are transported; ACN transport significantly increased in the presence of ethanol; Cells pre-treated with ACNs showed *ca*. 50% increased transport; GLUT2 may be responsible for ACN transport	[42]
Açaí pulp	Chemical extraction	Cy-3-Rut, Cy-3-Glu	50–500 μg/mL	ATCC	21 days	30–120 min	Transport efficiency of ACNs was 0.5%–4.9%; Presence of polymeric ACNs decreased transport of monomeric ACN glycosides (up to 40.3%)	[43]
Standard	-	Cat-Mv-3-Glu, Mv-3-Glu	100 μM	n/a	21 days	30–120 min	Transport efficiency of Mv-3-Glu was 4%; Absorption efficiency of Cat-Mv-3-Glu was lower than Mv-3-Glu (*ca*. 3%)	[44]
Sour cherry fruit and nectar	Chemical extraction	Cy-3-Glu-Rut	55 μM	ATCC	23–24 days	360 min	Cy-3-Glu-Rut recovery was *ca*. 0.5%–4%; Cy-3-Glu-Rut transported 3 times more efficiently from nectar than fruit; Sucrose and citric acid enhanced the transport of Cy-3-Glu-Rut (*ca*. 5-fold); SPE reduced the transport efficiency of Cy-3-Glu-Rut by 5–10-fold	[11]
Standard	Encapsulation	Cy-3-Glu	37.5 μM	n/a	20–26 days	60 min	Nano-encapsulated Cy-3-Glu with apoferritin was more efficiently transported compared to free Cy-3-Glu	[29]
Standard	-	Cy-3-Glu	10–40 μM	ATCC	13 days	30–120 min	Transport efficiency of Cy-3-Glu was 0.8%–2.4%; Phloridzin and phloretin inhibited the absorption of Cy-3-Glu; SGLT1 and GLUT2 are probably involved in the absorption of Cy-3-Glu	[45]
Açaí concentrate	Chemical extraction	Cy-3-Glu, Cy-3-Rut	500 μg/mL	ATCC	18–21 days	0–120 min	Transport rate of ACNs was 1.22%; Phospholipids from soy lecithin and terpenes from cold pressed citrus oil increased the transport of ACNs	[46]
Strawberry	Chemical extraction + *in vitro* digestion	Pg-3-Glu, Pg-3-Mal-Glu, Cy-3-Glu	16.3 mg/100 g	ATCC	21 days	120 min	Trace amount of Pg-3-Glu was transported	[47]
Grape	Chemical extraction	Mv-3-Glu, Pn-3-Glu, Pt-3-Glu, Cy-3-Glu, Dp-3-Glu	1766.1 μg/mL	ATCC	21 days	30–240 min	Mv-3-Glu, Pn-3-Glu, Pt-3-Glu and Cy-3-Glu were transported, whereas Dp-3-Glu was not transported; Transport efficiency of major anthocyanin (Mv-3-Glu) was 0.35%	[48]
Grape/blueberry extract	-	Mv-3-Glu, Pn-3-Glu, Pt-3-Glu, Dp-3-Glu, Cy-3-Glu, Mv-3,5-DGlu, Pn-3,5-DGlu	2613 μM	ATCC	21 days	0–90 min	Absorption rates of Mv-3-Glu, Pn-3-Glu, Pt-3-Glu, Dp-3-Glu and Cy-3-Glu were 0.005%–0.06%; Mv-3,5-DGlu and Pn-3,5-DGlu were not transported in quantifiable concentrations	[49]

ACN: anthocyanin; ATCC: American type culture collection; Cy-3-Gal: cyanidin-3-galactoside; Cy-3-Glu: cyanidin-3-glucoside; Cy-3-Glu-Rut: cyanidin-3-glucosylrutinoside; Cy-3-Rut: cyanidin-3-rutinoside; Dp-3-Glu: delphinidin-3-glucoside; Dp-3-Rut: delphinidin-3-rutinoside; DSMZ: German collection of microorganisms and cell cultures; GLUT2: glucose transporter 2; LY: Lucifer yellow; Mv-3-Glu: malvidin-3-glucoside; Mv-3,5-DGlu: malvidin-3,5-diglucoside; n/a: not available; Pg-3-Glu: pelargonidin-3-glucoside; Pg-3-Mal-Glu: pelargonidin-3-malonyl-glucoside; Pn-3-Gal: peonidin-3-galactoside; Pn-3-Glu: peonidin-3-glucoside; Pn-3,5-DGlu: peonidin-3,5-diglucoside; Pt-3-Glu: petunidin-3-glucoside; SGLT1: sodium-dependent glucose transporter 1; SPE: solid phase extraction; TEER: trans epithelial electrical resistance.

Few studies compared the transport efficiency of anthocyanins across Caco-2 cells with other polyphenols. The transport of both Mv-3-Glu and catechin through Caco-2 cells was found to be time dependent and reached approximately to the same value (4%) after 120 min of incubation [44]. Similarly, the recovery of epicatechin in the basolateral side (1%–4%) was also about the same with Cy-3-Glu-Rut (0.5%–4%) [11]. Reported transport of some other flavonoids through Caco-2 cells was 30% for quercetin, 17% for genistein and 6% for epicatechin [55].

The aglycone structure of anthocyanins is one of the many factors influencing their transport. For instance, Dp-3-Glu from blueberry extract showed lower transport efficiency compared to Mv-3-Glu and Pn-3-Glu. This may be a result of the higher number of hydroxyl groups in Dp or the greater hydrophobic structure of Mv that facilitated an increased portioning into cells and tissues. In addition, Dp has no OCH_3_ group, while Pn has one and Mv has two OCH_3_ groups (Figure 1), indicating that hydrophilic and hydrophobic groups affect the absorption of anthocyanins [28]. Similarly, for black currant anthocyanins the loss of delphinidins was significantly higher than cyanidins. Thus, the structural features might be crucial for anthocyanin stability [41].

Sugar moieties and polymeric structures may also have an influence on anthocyanin absorption by Caco-2 cells. For blueberry extracts, glucose-based anthocyanins had higher bioavailability than galactose-based anthocyanins. [28]. On the other hand, for black currant anthocyanins no differences are shown between the respective glucose and rutinose sugar moieties indicating that sugar conjugates may have a minor effect on anthocyanin stability [41]. The presence of polymeric anthocyanins in açaí fruit decreased the transport of monomeric anthocyanins glycosides in a dose-dependent manner by up to 40.3% [43]. Similarly, the absorption efficiency of flavanol-anthocyanin dimer Catechin-Mv-3-Glu, an anthocyanin derivative reported in grape skins and red wine, was lower than Mv-3-Glu (*ca.* 3%) [44].

The presence of other food components has been shown to have a major impact on anthocyanin transport. Solid phase extraction (SPE) of sour cherry extracts reduced the transport efficiency of Cy-3-Glu-Rut by 5–10-fold [11]. Ethanol, one of the main constituents of red wine, improved anthocyanin transport through Caco-2 cells [42]. However, this hypothesis is open to debate as there are some *in vivo* reports [56,57] claiming that ethanol has no influence on anthocyanin absorption. The ethanol concentration used in the cell culture study (1%), which was non-toxic to Caco-2 cells [42], is much lower than the actual ethanol concentration in red wine. Therefore the impact of ethanol on anthocyanin absorption and bioavailability may depend on the models as well as the doses used [42]. Citric acid also enhanced anthocyanin transfer to the basolateral side of Caco-2 cells [11]. This may be linked to the fact that anthocyanins are more stable at low pH values [16,58] (Figure 2). This effect of pH on transport across Caco-2 cells may have some physiological relevance. Although the cellular interstices and blood have a pH of around 7.4, the pH in the upper gastrointestinal tract under fasting conditions ranges from 5.0 to 6.5. In addition, the pH of the acidic microclimate just above the epithelial cell layer has been reported to be between 5.8 and 6.3 [11,28]. Furthermore, phospholipids from soy lecithin and terpenes from cold pressed citrus oil increased the transport of açaí anthocyanins in an *in vitro* cell monolayer model with Caco-2 cells, and a combination of phospholipids and terpenes was found to be the most effective [46].

In addition to the factors mentioned above, the physiological pH and temperature conditions (pH 7 and 37 °C) used in Caco-2 cell culture experiments may have a great influence on the stability of anthocyanins. In fact, a study on Cy-3-Glu [59] showed that there was no significant difference between the Caco-2 cell and cell free incubations in terms of the losses of Cy-3-Glu and the appearance of degradation products. These findings suggest that the loss of anthocyanins may be the result of spontaneous chemical breakdown rather than Caco-2 cell induced enzymatic deglycosylation followed by chemical degradation.

Although the exact mechanism of anthocyanin absorption in the small intestine is still unclear, it has been proposed that anthocyanins could interfere with the transporters responsible for their own transport. The candidates for anthocyanin transporters were the glucose transporters, since anthocyanins possess a sugar moiety, in particular a glucose residue. SGLT1 and GLUT2 are the main hexose transporters described in Caco-2 cells. SGLT1 is an energy-dependent and sodium-dependent cotransporter, whereas GLUT2 is a facilitated transporter. SGLT1 is only present on the apical membrane and until a few years ago, GLUT2 was described to be present only in the basolateral membrane and in some pathologies on the apical membrane. Recently, it has been described and accepted that GLUT2 is present on the apical side and can be gathered to the membrane in the presence of a large amount of glucose, therefore becoming the main transporter responsible for glucose uptake [42,45]. It was found that GLUT2 expression assessed by RT-PCR was increased in Caco-2 cells pretreated with red grape skin anthocyanins, by comparison with controls, indicating that chronic consumption of anthocyanins could be favorable for their own bioavailability. In addition, the tested red grape skin anthocyanins interfered with glucose uptake resulting in an inhibitory effect (about 60% decrease) [42]. Similarly, Pn-3-Glu from strawberry extract was able to influence glucose uptake into the cells and transport to the basolateral side by inhibiting activities of the glucose transporters [27]. Another study also confirmed that exposure to anthocyanin rich berry extract derived from blueberry, bilberry, cranberry, elderberry, raspberry seeds and strawberry significantly reduce SGLT1 and GLUT2 expressions [60]. Inhibition studies conducted using the pharmacological agents, phloridzin, an inhibitor of SGLT1, or phloretin, an inhibitor of GLUT2, revealed that the absorption of Cy-3-Glu was significantly inhibited in the presence of these agents [45]. These data suggest that anthocyanins may prevent hyperglycemia by decreasing glucose transporter expressions.

Since the high instability of anthocyanins has a direct impact on their potential health benefits, food processing technologies such as encapsulation may be used to improve their bioavailability [61,62,63]. Accordingly, the nano-encapsulated Cy-3-Glu with apoferritin was more efficiently transported through a Caco-2 cell monolayer compared to free Cy-3-Glu [29]. In another study, processing of sour cherry fruit into nectar led to three times more efficient transport of Cy-3-Glu-Rut through a Caco-2 cell monolayer [11].

### 3.3. Anthocyanin Metabolism by Caco-2 Cells

**Figure 3:**
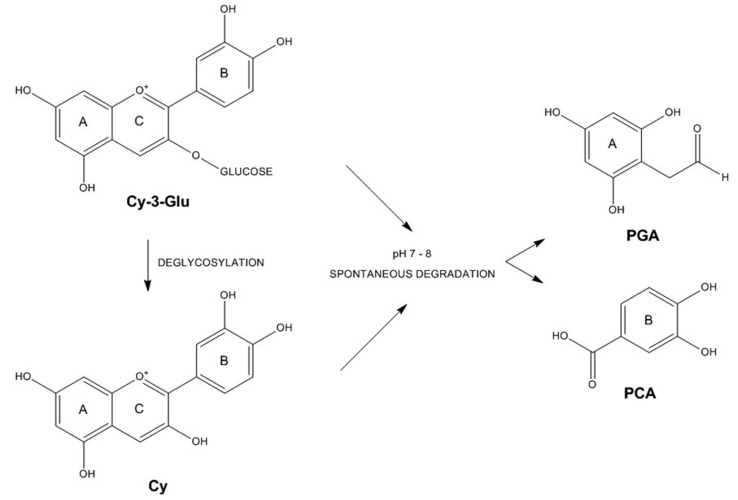
Metabolites (PGA: phloroglucinaldehyde; PCA: protocatechuic acid) of cyanidin-3-glucoside (Cy-3-Glu) and cyanidin (Cy).

Polyphenols undergo Phases I and II transformations in the human body. Phase I transformations consist of oxidation, reduction and hydrolysis, but these transformations occur less frequently. Phase II biotransformations taking place in the liver and the intestine occur more intensively. These Phase II transformations consist of conjugation reactions where different are formed (methyl, glucuronic and sulfate derivatives) [64]. Spontaneous transformation of anthocyanins to phenolic acids and aldehydes is reported to occur under biological conditions [65]. Confirming that, under cell culture conditions, the main metabolites of Cy-3-Glu and Cy are detected as protocatechuic acid (PCA) and phloroglucinaldehyde (PGA), which are derived from the A and B rings of the parental compound (Figure 3). With action of enzymes, these metabolites can be further degraded to glucuronide and sulfate conjugates [59]. Another important Phase II reaction of anthocyanins is the methylation, which alters the number of hydroxyl and methoxyl groups in ring B in comparison with the native compound. Although not so intense as genuine anthocyanins, methylated metabolites of Cy-3-Glu, Dp-3-Glu and Pt-3-Glu displayed some antiproliferative activity for the Caco-2 cell line [66]. On the other hand, some other anthocyanin metabolites including gallic acid, 3-*O*-methylgallic acid, and PGA reduced cell proliferation in Caco-2 cells more effectively compared to parental anthocyanins [67]. Therefore when assessing the health benefits of anthocyanins, potential effects of such metabolites should be taken into account.

### 3.4. Bioactive Properties of Anthocyanins on Caco-2 Cells

Although the bioavailability of anthocyanins is very low, anthocyanins from several different food sources have been shown to exert health-promoting effects on Caco-2 cells (Table 2). These potential bioactive properties included antiproliferative [68,69,70,71,72,73,74,75,76,77,78,79], antioxidant [79,80,81,82,83,84,85] and anti-inflammatory [48,86,87] effects. MTT (3-(4,5-dimethylthiazolil-2-yl)-2,5-diphenyl-tetrazoliumbromide) is the most common assay that is applied to evaluate the antiproliferative effects of anthocyanins on Caco-2 cells [68,69,73,74,76,77,78]. This assay is based on the conversion of the yellow tetrazolium salt MTT by mitochondrial dehydrogenase of live cells to the purple formazan. Trypan blue [70,79], thymidine incorporation [71], CCK-8 (cell counting kit 8) [72], LDH (lactate dehydrogenase) [73] and SRB (sulphorhodamine B) [74] are some other assays that are used to determine the cell viability after anthocyanin treatment. For the antioxidant activity, the formation of intracellular ROS (reactive oxygen species) is often determined using a fluorescent probe, DCFH-DA (2′,7′-dichloro-dihydro-fluorescein diacetate) [79,80,81,82,84,85]. Prior to anthocyanin treatment, the oxidation is initiated with different compounds including t-BHP (*tert*-butyl hydroperoxide) [79,80,85] and AAPH (2,2′-azobis (2-amidinopropane) dihydrochloride) [81,82]. Anti-inflammatory activities of anthocyanins included reduction of NF-κB (nuclear factor κB) activation [86,87], inhibition of NO (nitric oxide) secretion [87], downregulating the expression of pro-inflammatory cytokines (particularly IL-8) and reducing the levels of adhesion molecules [48].

## 4. Conclusions and Future Perspectives

Currently, the Caco-2 cell line is considered to be the most common *in vitro* model of the small intestine, despite some heterogeneity in its characteristics and some other limitations arising from its tumoral origin. A major difference between the Caco-2 cells and the intestinal enterocytes is that the Caco-2 cells do not have a mucus layer. Studies have been performed to co-culture a mucin secreting cell line (HT-29) with the Caco-2 cell line, but they did not give the expected results. Another limitation of using Caco-2 cells is the poor reproducibility of results between different laboratories. Thus, a standardization of some important parameters such as cell origin, passage number and incubation time is necessary. On the other hand, there are also some benefits of using this cell line while evaluating anthocyanin absorption. It is a high throughput model, which allows the screening of a large number of samples. Furthermore, the use of the Caco-2 cell model is also important to study molecular mechanisms of anthocyanin absorption. When the transport of anthocyanins is assessed using Caco-2 cells as intestinal models, in almost all studies cells are treated with pure standards or anthocyanin-rich extracts derived from plants and foods and data are reported at concentrations that showed a response. However, plasma and tissues are not exposed *in vivo* to anthocyanins in these forms. In this sense, the use of combined *in vitro* digestion and Caco-2 cells could be a better approach. Also, the anthocyanin concentrations tested should be of the same order as the maximum plasma concentrations attained after a polyphenol-rich meal, which are in the range of 0.1–10 μmol/L [88]. In future studies, these conditions should be taken into account and the methodologies should be adopted accordingly.

Studies investigating anthocyanin absorption by Caco-2 cells reported very low transport of these compounds. The observed trends among different anthocyanins generally agreed with the published *in vivo* results. In spite of convincing observations in Caco-2 cell culture model, extrapolation of these *in vitro* findings for anthocyanins to the *in vivo* situation is difficult due to the unknown accumulation of these compounds at target tissues. Besides, the bioavailability of anthocyanins may be underestimated both *in vitro* and *in vivo* since the metabolites formed in the course of digestion could be responsible for the health benefits associated with anthocyanins. Recently, it was suggested that anthocyanins could also be absorbed from the stomach. In cell culture studies, anthocyanins were found to be able to cross MKN-28 cell monolayers (differentiated adenocarcinoma stomach cells) [89,90,91]. Therefore, the existing knowledge indicates that the observed low apparent bioavailability of anthocyanins could be due to their extensive presystemic metabolism, rather than poor absorption from the intestinal lumen. In addition, some anthocyanins can reach the colon in significant amounts and undergo microbial fermentation. The resultant microbial metabolites may also contribute to the health effects of anthocyanins. Eventually, we suggest that future studies should address the bioavailability of the anthocyanin metabolites to establish whether such metabolites could play a part in bioactivity.

**Table 2:** Bioactive properties of anthocyanins on Caco-2 cells.

Bioactivity	Sources	Assays/Markers	Anthocyanins	References
Antiproliferative	Arctic bramble, Black currant, Blueberry, Bilberry, Chokeberry juice, Cloudberry, Lingonberry, Peach, Plum, Potato, Purple rice, Red chicory, Standards, Strawberry, Strawberry guava	MTT, Trypan blue, Thymidine incorporation, CCK-8, LDH, SRB	Cy, Cy-3-Ara, Cy-3-Gal, Cy-3-Glu, Cy-3-Rut, Dp, Dp-3-Gal, Dp-3-Glu, Dp-3-Rut, Mv-3-Ara, Mv-3-Gal, Mv-3-Glu, Pg, Pn-3-Gal, Pn-3-Glu, Pt-3-Gal, Pt-3-Glu	[68,69,70,71,72,73,74,75,76,77,78,79]
Antioxidant	Bee pollen, Bilberry, Blackberry, Red chicory, Red orange, Wine	ROS, TBARS	Cy-3-Ara, Cy-3-Gal, Cy-3-Glu, Dp-3-Ara, Dp-3-Gal, Dp-3-Glu, Dp-3-Rut, Mv-3-Ace-Glu, Mv-3-Ara, Mv-3-Caf-Glu, Mv-3-Cou-Glu, Mv-3-Gal, Mv-3-Glu, Mv-3-Rut, Pn-3-Ara, Pn-3-Cou-Glu, Pn-3-Gal, Pn-3-Glu, Pt-3-Ara, Pt-3-Cou-Glu, Pt-3-Gal, Pt-3-Glu, Pt-3-Rut	[79,80,81,82,83,84,85]
Anti-inflammatory	Blackberry, Blueberry, Black raspberry, Grape, Raspberry	NF-κB, NO, IL-8, E-selectin, ICAM-1, VCAM-1	Cy-3-Ara, Cy-3-Gal, Cy-3-Glu, Cy-3-Rut, Dp-3-Ara, Dp-3-Gal, Dp-3-Glu, Mv-3-Ara, Mv-3-Gal, Mv-3-Glu, Pn-3-Gal, Pn-3-Glu, Pt-3-Ara, Pt-3-Gal, Pt-3-Glu	[48,86,87]

CCK-8: cell counting kit 8; Cy: cyanidin; Cy-3-Ara: cyanidin-3-arabinoside; Cy-3-Gal: cyanidin-3-galactoside; Cy-3-Glu: cyanidin-3-glucoside; Cy-3-Rut: cyanidin-3-rutinoside; Dp: delphinidin; Dp-3-Gal: delphinidin-3-galactoside; Dp-3-Ara: delphinidin-3-arabinoside; Dp-3-Glu: delphinidin-3-glucoside; Dp-3-Rut: delphinidin-3-rutinoside; ICAM-1: intercellular cell adhesion molecule-1; IL-8: interleukin 8; LDH: Lactate dehydrogenase; MTT: 3-(4,5-dimethylthiazolil-2-yl)-2,5-diphenyl-tetrazoliumbromide; Mv-3-Ace-Glu: malvidin-3-acetylglucoside; Mv-3-Ara: malvidin-3-arabinoside; Mv-3-Caf-Glu: malvidin-3-trans-caffeoyglucoside; Mv-3-Cou-Glu: malvidin-3-trans-p-coumarylglucoside; Mv-3-Gal: malvidin-3-galactoside; Mv-3-Glu: malvidin-3-glucoside; Mv-3-Rut: malvidin-3-rutinoside; NF-κB: nuclear factor κB; NO: nitric oxide; Pg: pelargonidin; Pn-3-Ara: peonidin-3-arabinoside; Pn-3-Cou-Glu: peonidin-3-trans-p-coumarylglucoside; Pt-3-Cou-Glu: petunidin-3-p-coumarylglucoside; Pn-3-Gal: peonidin-3-galactoside; Pn-3-Glu: peonidin-3-glucoside; Pt-3-Ara: petunidin-3-arabinoside; Pt-3-Gal: petunidin-3-galactoside; Pt-3-Glu: petunidin-3-glucoside; Pt-3-Rut: petunidin-3-rutinoside; ROS: reactive oxygen species; SRB: sulphorhodamine B; TBARS: thiobarbituric acid reactive substances; VCAM-1: vascular cell adhesion molecule-1.
